# 2D Transition Metal Dichalcogenides and Graphene-Based Ternary Composites for Photocatalytic Hydrogen Evolution and Pollutants Degradation

**DOI:** 10.3390/nano7030062

**Published:** 2017-03-15

**Authors:** Ying Chen, Hongqi Sun, Wenchao Peng

**Affiliations:** 1 School of Chemical Engineering and Technology, Tianjin University, Tianjin 300072, China; yingchen@tju.edu.cn; 2 School of Engineering, Edith Cowan University, 270 Joondalup Drive, Joondalup, Perth, WA 6027, Australia; 3 Department of Chemical Engineering, Renai College of Tianjin University, Tianjin 301636, China

**Keywords:** transition metal dichalcogenides (TMDs), graphene, photocatalytic, hydrogen evolution, pollutants degradation

## Abstract

Photocatalysis have attracted great attention due to their useful applications for sustainable hydrogen evolution and pollutants degradation. Transition metal dichalcogenides (TMDs) such as MoS_2_ and WS_2_ have exhibited great potential as cocatalysts to increase the photo-activity of some semiconductors. By combination with graphene (GR), enhanced cocatalysts of TMD/GR hybrids could be synthesized. GR here can act as a conductive electron channel for the transport of the photogenerated electrons, while the TMDs nanosheets in the hybrids can collect electrons and act as active sites for photocatalytic reactions. This mini review will focus on the application of TMD/GR hybrids as cocatalysts for semiconductors in photocatalytic reactions, by which we hope to provide enriched information of TMD/GR as a platform to develop more efficient photocatalysts for solar energy utilization.

## 1. Introduction

Since the discovery of the photocatalytic splitting of water on TiO_2_ electrodes by Fujishima and Honda in 1972 [1], photocatalysis has attracted great attention for eliminating hazardous pollutants and generating sustainable energy [2]. Semiconductors such as TiO_2_, ZnO, CdS, etc. can act as photocatalysts for the utilization of solar energy [3]. They are however limited in real application by the rapid electron–hole recombination [4,5,6]. Noble metal cocatalysts are usually loaded to enhance the activity of the semiconductor photocatalysts [7]. However, these metals are rare and expensive to apply [8]. The development of highly active and low cost cocatalysts remains a great challenge in the field of photocatalysis.

Transition metal dichalcogenides (TMDs) such as MoS_2_ and WS_2_ have exhibited excellent activities as cocatalysts for the modification of semiconductors [8,9]. The properties of TMDs can be tailored according to their crystalline structure and the number and stacking sequence of their nanosheets [10,11,12,13,14]. By loading TMDs cocatalysts, semiconductor–semiconductor or metal–semiconductor junctions will form, and more interfaces could be created [15,16]. Charge separation and electron transport can therefore be enhanced, leading to the activity improvement [15]. Furthermore, many kinds of TMDs with different phases were reported to be active for the electrochemical hydrogen evolution reaction (HER), which stems from their exposed and under-coordinated edge sites [17,18,19]. Therefore, loading the TMDs as cocatalysts for semiconductors could also lower the activation energy and overpotential for photocatalytic H_2_ evolution [8]. On the other hand, TMDs have special 2D layered structure, and can be used as effective supports for anchor of semiconductor nanoparticles, which could reduce the mobility, provide more active sites, and avoid coalescence and agglomeration of the semiconductors [20,21,22]. Based on the above analysis, TMDs have shown great potential as substitute of noble metal cocatalysts for the synthesis of composite photocatalysts with high activity [8]. 

Graphene (GR) consists of a single layer and sp^2^-hybridized carbon lattice with excellent electrical (200,000 cm^2^·V^−1^·s^−1^), thermal, and mechanical properties, and is a novel material that has emerged as a rapidly rising star in the field of material science [23]. The photocatalytic activity of semiconductors can be greatly increased by loading GR as cocatalyst, mainly owing to the effective separation of the electron–hole pairs [24,25]. 

By combining GR with TMDs, new hybrid cocatalysts could be synthesized with 2D layered structures. GR here can transport the photogenerated electrons rapidly, and the TMDs in the hybrids can accept electrons and act as active sites for H_2_ evolution or radicals generation. This mini review will focus on the synthesis methods of TMD/GR-based photocatalysts and their applications for photocatalytic H_2_ evolution and organic pollutants degradation. Based on this review, we hope to offer enriched information of TMD/GR as a platform to fabricate more efficient photocatalysts for solar energy utilization.

## 2. TMD/GR-Based Composites for Photocatalytic H_2_ Evolution

**Figure 1:**
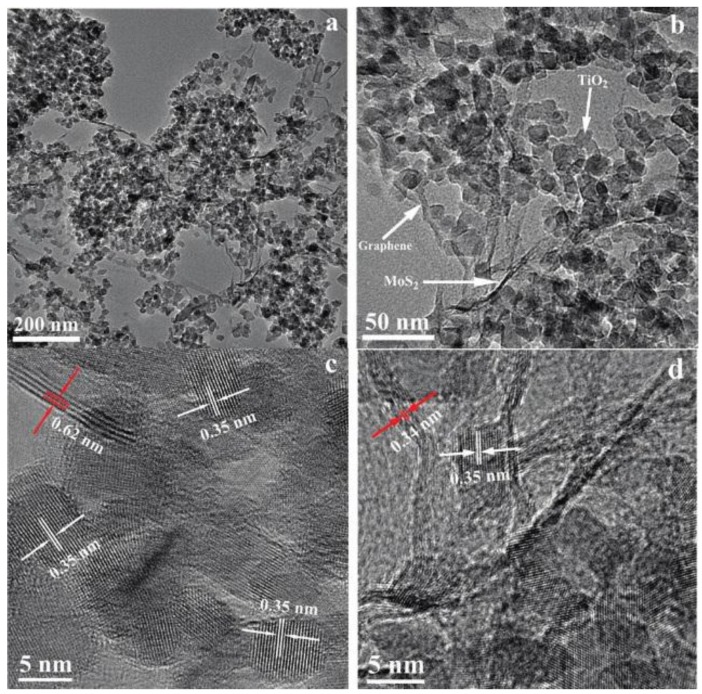
Morphology characterization of the TiO_2_-MoS_2_/graphene (GR) composite. (**a**,**b**) Transmission electron microscopy (TEM) and (**c**,**d**) high-resolution TEM (HRTEM) images of the TiO_2_-MoS_2_/GR composite (reprinted from [25] with permission, Copyright American Chemical Society, 2012).

TiO_2_ is the most frequently used semiconductor for photocatalytic H_2_ evolution, which can only absorb and utilize UV light due to its large band gap (3.2 eV) [26]. Xiang et al. synthesized ternary composites consisting of TiO_2_ nanoparticles grown on the MoS_2_/GR hybrid as enhanced photocatalysts for H_2_ evolution (Figure 1) [25]. The TiO_2_-MoS_2_/GR composites were prepared using a two-step hydrothermal method. As shown in Figure 1, the TiO_2_ nanoparticles were supported on the 2D MoS_2_/GR hybrid uniformly with intimate contact. The electrons can therefore transfer rapidly from TiO_2_ to the MoS_2_/GR cocatalyst, and the charge recombination can therefore be suppressed. The activity of the ternary composites can be tuned by adjusting the GR percentage of MoS_2_/GR cocatalysts and the percentage of the MoS_2_/GR hybrid for the ternary photocatalysts. The optimized TiO_2_-MoS_2_/GR composite could obtain a high H_2_ evolution rate of 165.3 μmol·h^−1^ and a quantum efficiency of 9.7% at 365 nm. 

**Figure 2:**
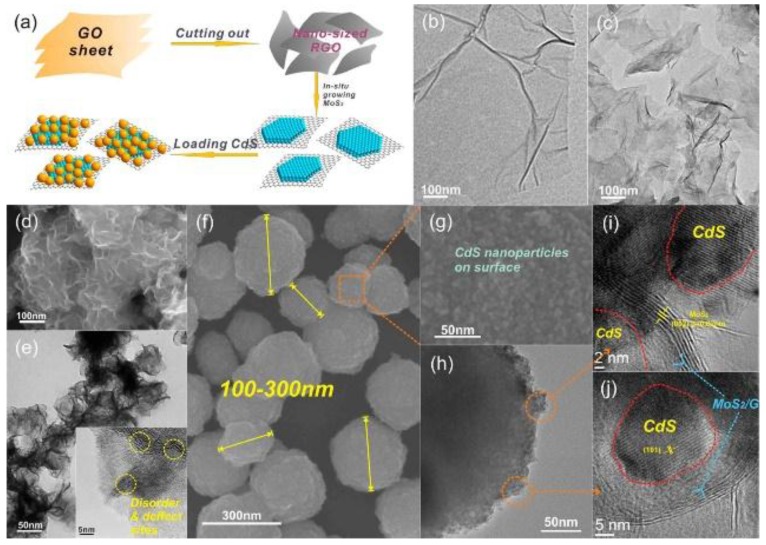
(**a**) Schematic illustration of growth mechanism of MoS_2_/GR-CdS composites; TEM images of (**b**) graphene oxide (GO) and (**c**) nanosized graphene (GR); (**d**) Scanning electron microscopy (SEM) and (**e**) TEM images of as-prepared MoS_2_/GR composite; the inset of (**e**) is the HRTEM image of MoS_2_/GR composite; (**f** and **g**) SEM images of CdS-MoS_2_/GR composites; (**h**) TEM and (**i** and **j**) HRTEM images of the CdS-MoS_2_/GR composite (reprinted from [27] with permission, Copyright American Chemical Society, 2014).

**Figure 3:**
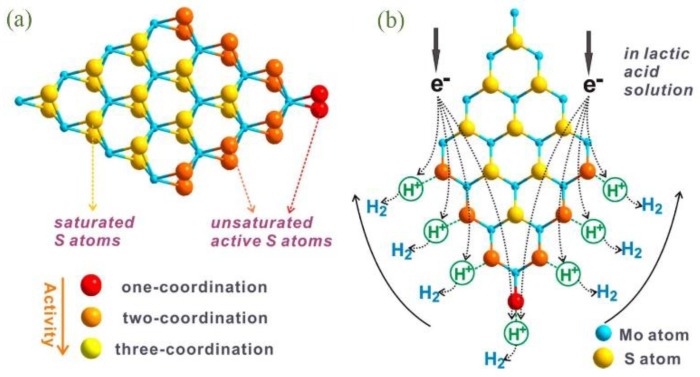
(**a**) Schematic illustration of the microstructure of MoS_2_ and (**b**) its cocatalytic mechanism of H_2_ generation in lactic acid solution (reprinted from [27] with permission, Copyright American Chemical Society, 2014).

CdS has a narrow bandgap of 2.3 eV, which is effective for capturing the visible light [36]. Chang et al. used nanosized GR as support for the growth of MoS_2_; 3D hierarchical CdS-MoS_2_/GR composites with diameters of 100–300 nm were then synthesized with the help of polyvinylpyrrolidone (PVP) (Figure 2) [27]. As shown in Figure 2e, the MoS_2_ sheets on nanosized GR have many defect sites and disordered structures due to the low synthesis temperature, CdS nanoparticles can then firmly anchor on these defects and vacancies (Figure 2g–j). After optimizing each component proportion, the highest H_2_ evolution rate could be as large as 1.8 mmol/h with an apparent quantum efficiency (AQE) of 28.1% at 420 nm, which was even higher than that of Pt/CdS. They thought that the activities of S atoms in the MoS_2_ molecules were different with respect to their different coordination (Figure 3a). Unsaturated S atoms are active for H^+^ adsorption and reduction (Figure 3b), while the saturated S atoms on the basal plane are inert. The nanosized few-layer MoS_2_ supported on GR has more exposed edges and unsaturated active S atoms, which is therefore a promising cocatalyst for the activity enhancement of CdS.

**Figure 4:**
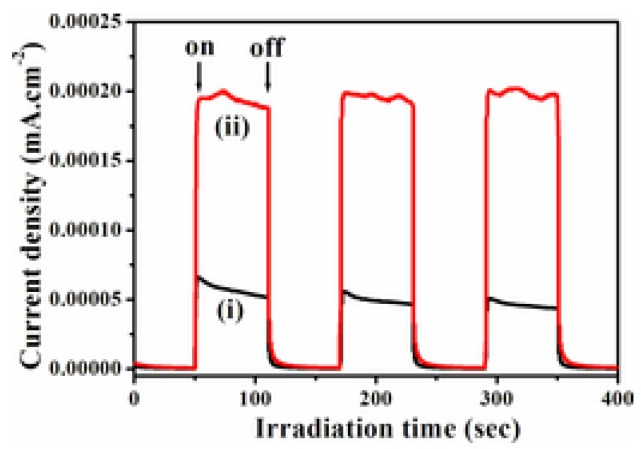
Transient photocurrent responses of pure CdS and CdS-WS_2_/GR composite in 1 M Na_2_SO_4_ aqueous solution under visible-light irradiation at 0.5 V versus Ag/AgCl (reprinted from [32] with permission, Copyright Wiley-VCH, 2016).

Xiang et al. synthesized CdS-WS_2_/GR ternary composites for photocatalytic H_2_ evolution [32]. The optimized WS_2_/GR content in the ternary CdS-WS_2_/GR composites was determined to be 4.2 wt %. Using 0.35 M Na_2_S/0.25 M Na_2_SO_3_ as sacrificial agent, a satisfactory H_2_ evolution rate of 1.842 mmol·h^−1^·g^−1^ could be achieved with an apparent quantum efficiency of 21.2% at 420 nm. The transient photocurrent response was also enhanced by loading the WS_2_/GR cocatalyst, which was promising evidence for the improved charge transport (Figure 4). By loading WS_2_/GR cocatalyst, more active sites will be introduced, and charge separation and interfacial charge transfer could be enhanced, thus leading to the greatly increased photo-activity.

**Figure 5:**
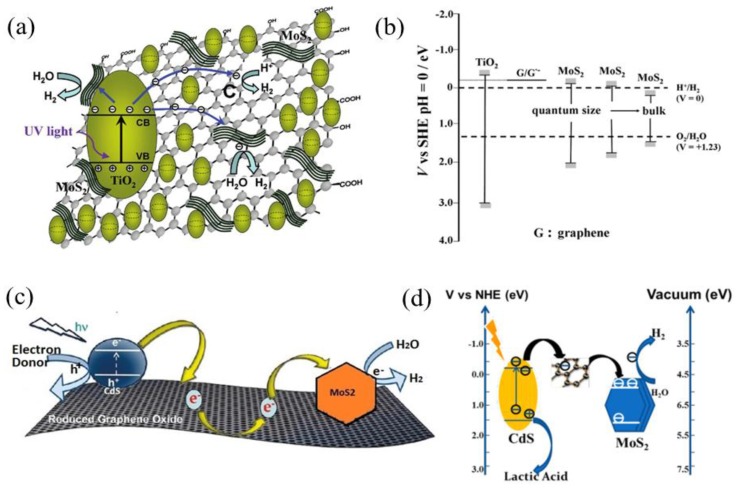
Schematic illustration of (**a**) the charge transfer in TiO_2_-MoS_2_/GR composites for photocatalytic H_2_ evolution; and (**b**) the potential and band positions in the TiO_2_/MoS_2_/graphene system (reprinted from [25] with permission, Copyright American Chemical Society, 2012); (**c**) Graphene-supported CdS and MoS_2_ for photocatalytic hydrogen evolution; (**d**) The band positions for the CdS–graphene–MoS_2_ system (reprinted from [28] with permission, Copyright Royal Society of Chemistry, 2014). (SHE: Standard hydrogen electrode; NHE: Normal hydrogen electrode).

During the photocatalytic H_2_ evolution process, the metal oxide can absorb a photon to create an electron–hole pair with irradiation (Figure 5a,c). Both of the graphene/ graphene^•−^ redox potential and conduction band (CB) of quantum-sized MoS_2_ are slightly lower than the CB of anatase TiO_2_ or CdS (Figure 4b,d). Part of the excited electrons can then directly transfer to active sites of MoS_2_, and another part transfer to active sites of MoS_2_ via graphene conducting channel. Graphene here can act as a “highway” for the rapid transport of photo-generated electrons, while MoS_2_ nanosheets can accept electrons and act as active sites for H_2_ evolution. Therefore, using MoS_2_/GR hybrid as cocatalyst, suppression of charge recombination, improvement of interfacial charge transfer, and an increase in the number of active sites could be achieved, thus leading to the enhanced photo-activity. 

It has been reported that sub-10 nm rutile TiO_2_ with 1 wt % Pt doping exhibited state-of-the-art activity among TiO_2_-based composites for photocatalytic water splitting. The hydrogen evolution rate could be achieved to 932 mmol·h^−1^·g^−1^ under visible light (>400 nm) and 1954 mmol·h^−1^·g^−1^ under simulated solar light [37]. By loading 0.30 wt % of Pt and 0.13 wt % of PdS as cocatalysts on CdS, another CdS-based state-of-the-art material could be synthesized with a quantum efficiency (QE) up to 93% and a hydrogen evolution rate of 8.77 mmol·h^−1^ [38]. Compared to these state-of-the-art materials, the activities of TMD/GR modified semiconductors are relatively weak, with lower H_2_ evolution rates and QEs. Although the TMD/GR cocatalysts are more cost effective, deep studies are still needed to obtain higher efficiencies for real application. 

## 3. TMD/GR-Based Photocatalysts for Pollutants Degradation

**Figure 6:**
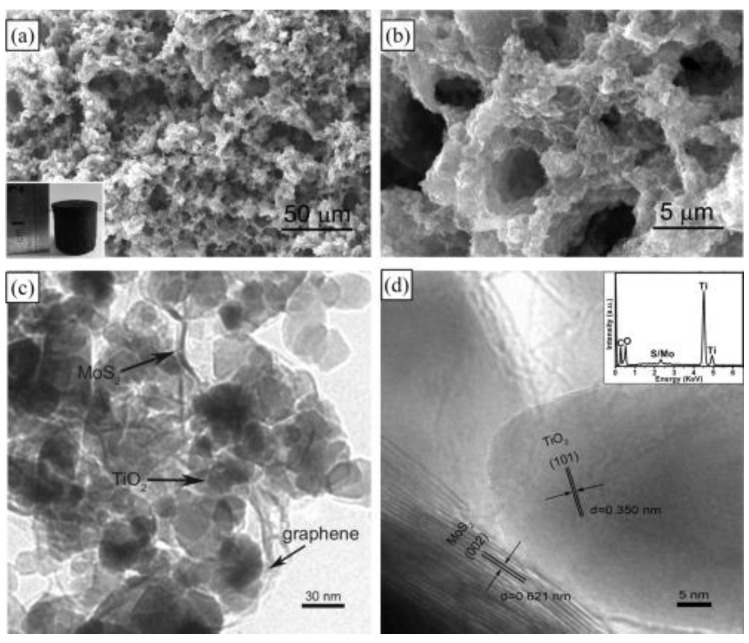
(**a**,**b**) SEM images of the as-prepared MoS_2_/P25/GR-aerogel; the inset image is a digital photo of the free-standing MoS_2_/P25/GR-aerogel. (**c**,**d**) TEM images and energy-dispersive X-ray spectroscopy (EDS) (insert) pattern of the as-prepared MoS_2_/P25/GR-aerogel (reprinted from [40] with permission, Copyright Elsevier, 2014).

**Figure 7:**
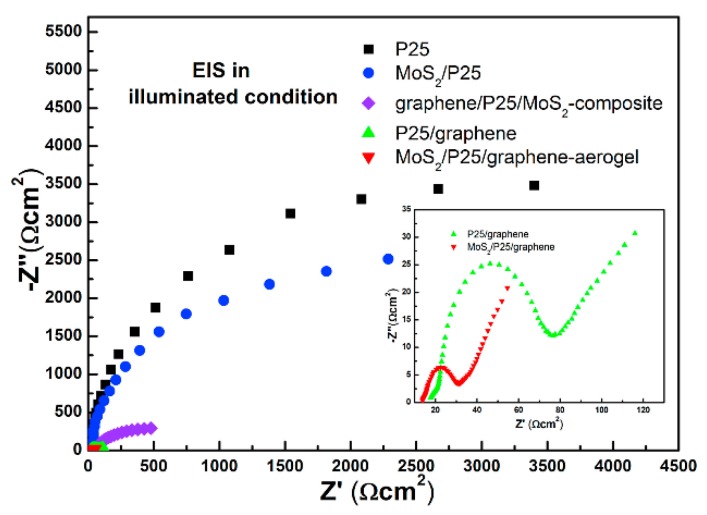
Electrochemical impedance spectroscopy (EIS) Nyquist plots of MoS_2_/P25/GR-aerogel, GR/P25/MoS_2_-composite, P25/GR, MoS_2_/P25, and P25 nanoparticles in sulfide-sulfite electrolyte and under UV irradiation (reprinted from [40] with permission, Copyright Elsevier, 2014).

**Figure 8:**
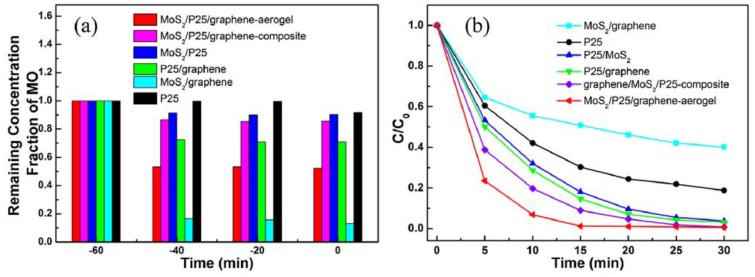
(**a**) Bar plot showing the remaining methyl orange (MO) in solution after reaching the adsorption equilibrium in the dark over MoS2/P25/GR-aerogel, GR/P25/MoS2-composite, P25/GR, MoS2/P25, MoS2/GR, and P25; (**b**) photo-degradation of MO by MoS2/P25/GR-aerogel, GR/P25/MoS2-composite, P25/GR, MoS2/P25, MoS2/GR, and P25 with a reaction time of 30 min under UV irradiation (reprinted from [40] with permission, Copyright Elsevier, 2014).

Photocatalysis is also an attractive technology for the degradation of pollutants in water using solar energy [39]. Han et al. used a hydrothermal method to combine the exfoliated MoS_2_, GR, and TiO_2_ P25 together [40]. The obtained composite was a novel graphene-based three-dimensional (3D) aerogel embedded with TiO_2_ particles and MoS_2_ nanosheets (Figure 6). Porous structure could be observed with pore sizes of about several micrometers (Figure 6b). The Nyquist plots of the samples were also tested, and the final 3D GR–MoS_2_–TiO_2_ composite had the smallest cure radius (Figure 7), indicating that the addition of 3D graphene aerogel can decrease the solid state interface layer resistance and the charge transfer resistance. During the application test, the final composite had better adsorption ability for methyl orange (MO) due to the maximization of accessible sites of the 3D interconnected networks (Figure 8a). The 3D photocatalyst was proved to be very effective for the photocatalytic degradation of MO, and nearly no MO was left after 15 min irradiation (Figure 8b).

**Figure 9:**
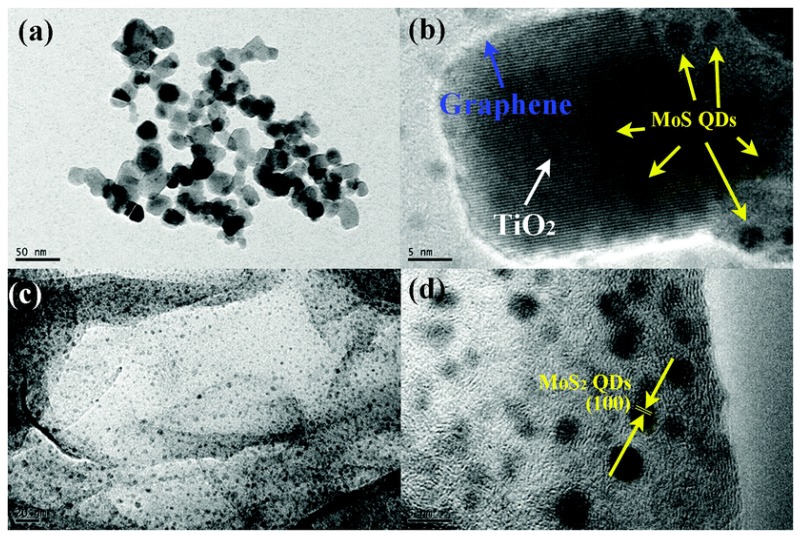
TEM and HRTEM images of the sample (**a**,**b**) TiO_2_-MoS_2_/GR and (**c**,**d**) MoS_2_–GR (reprinted from [34] with permission, Copyright Royal Society of Chemistry, 2015). QD: quantum dot.

Gao et al. fabricated a TiO_2_-MoS_2_/GR composite under atmospheric pressure using a simple one-pot solvothermal method [34]. Na_2_MoO_4_ and thiocarbamide were used as precursors for MoS_2_, and mixed solvent of (dimethylacetamide (DMAc)/deionized (DI) H_2_O) was used as reaction media. Under the above conditions, MoS_2_ quantum dots (QDs) with (100) face exposed could be generated on the surface of TiO_2_ and GR (Figure 9). Attributed to the small diameter of the MoS_2_ QDs, more active edge could be created, thus leading to the enhanced photocatalytic activity. 

Peng et al. synthesized Ag_3_PO_4_-MoS_2_/GR via a simple two-step hydrothermal process [33]. The composite was found to be an effective catalyst for the photo-decomposition of 2,4-dichlorophenol (DCP) under simulated solar light and visible light (λ > 420 nm). They described the major reaction steps involved in this photocatalytic process as follows:

Ag_3_PO_4_ + hv → Ag_3_PO_4_ (e^−^ + h^+^) (1)

Ag_3_PO_4_ (e^−^) + MoS_2_/GR → Ag_3_PO_4_ + MoS_2_/GR (e^−^) (2)

MoS_2_/GR (e^−^) + O_2_
→ MoS_2_/GR + O_2_^−^ (3)

Ag_3_PO_4_ (h^+^) + DCP → CO_2_ + H_2_O + other products (4)

Ag_3_PO_4_ (h^+^) + OH^−^ → Ag_3_PO_4_ + **·**OH (5)

**·**OH + DCP → CO_2_ + H_2_O + other products (6)

As shown in the mechanism, electrons and holes could be separated with irradiation (1). The holes could oxidize the DCP molecules adsorbed on the catalyst surface directly (4). They could also react with water (or hydroxyl) to form hydroxyl free radicals (**·**OH), which are strong oxidants for DCP decomposition (5). The MoS_2_/GR cocatalyst here could act as electron collectors to facilitate the interfacial electron transfer and charge separation. In addition, the MoS_2_/GR cocatalyst could also provide more active sites and allow for the activation of dissolved O_2_ for organic degradation [33]. 

Using CoS_2_/GR as cocatalyst, Zhu et al. supported TiO_2_ nanoparticles on its surface using a facile sonochemical and hydrothermal method [35]. Their photo-activity was then evaluated for the degradation of methylene blue (MB) and Texbrite BA-L (TBA) under visible light. Enhanced activity was obtained due to the synergetic effect between TiO_2_ and the CoS_2_/GR cocatalyst. The recent progress of TMD/GR based photocatalysts for H_2_ evolution and pollutants degradation are summarized and shown in Table 1 for a easier perusal.

## 4. Conclusions and Perspective

This mini review focused on the recent developments of the TMD/GR-based composites, including the synthesis methods, the application in photocatalytic H_2_ evolution, and the application for organic pollutants degradation. By combination with GR, the TMD/GR hybrids were more effective as cocatalysts for the modification of semiconductors. GR here can act as a conductive electron transport “highway” for the transport of the photogenerated electrons, and the TMDs nanosheets in the hybrids can accept electrons and act as active sites for photocatalytic reactions. Although deep research is still needed for real application, TMD/GR cocatalysts have shown great potential as a platform to fabricate more efficient photocatalysts for solar energy utilization.

## Figures and Tables

**Table 1:** Summary of transition metal dichalcogenides (TMD)/GR based photocatalysts and their applications.

Catalyst	Synthesis Method	Application	Light Source	Activity	Morphology	Ref.
TiO_2_-MoS_2_/GR	Two-step hydrothermal	H_2_ generation in 25% (*v*/*v*) ethanol/water	350 W Xe arc lamp 20 mW/cm^−2^ λ = 365 nm	165.3 μmol·h^−1^ ^a^ QE: 9.7% at 365 nm	Particles/sheets	[25]
CdS-MoS_2_/GR	Hydrothermal	H_2_ generation in 20 vol % lactic solution	300 W Xe lamp (λ > 420 nm)	1.8 mmol/h QE: 28.1% at 420 nm	Particles/sheets	[27]
CdS-MoS_2_/GR	Sonication assisted post loading	H_2_ generation in 10 vol % lactic acid	500 W UV-vis lamp	3.067 mL·h^−1^	Particles/sheets	[28]
CdS-MoS_2_/GR	In-situ photo deposition	H_2_ generation in 10 vol % lactic acid	350 W Xe lamp λ ≥ 420 nm 34 mW/cm^2^	99 μmol·h^−1^ QE: 9.8% at 420 nm	Particles/sheets	[29]
ZnS-MoS_2_/GR	One-pot hydrothermal	H_2_ generation in 0.005 M Na_2_S and 0.005 M Na_2_SO_3_	300 W Xe lamp 125 mW/cm^2^	2258 μmol·h^−1^·g^−1^	Particles/sheets	[30]
CdS-MoS_2_/GR	Two-step solvothermal	H_2_ generation in 10 vol % lactic acid	350 W xenon arc lamp (λ ≥ 420 nm)	621.3 μmol·h^−1^ 54.4% at 420 nm	Nanorods /sheets	[31]
CdS-WS_2_/GR	Solvothermal	H_2_ generation in 0.35 M Na_2_S and 0.25 M Na_2_SO_3_	500 W Xeno arc Lamp λ > 420 nm	1842 μmol·h^−1^·g^−1^ 21.2% at 420 nm	Nanosheets/nanorods	[32]
Ag_3_PO_4_-MoS_2_/GR	Hydrothermal-deposition	2,4-Dichlorophenol degradation 20 mg·L^−1^	500 W xenon lamp (λ > 420 nm)	^b^ DP of >99% in 20 min 25 times higher than N-TiO_2_	Sub-microcrystal/sheets	[33]
TiO_2_-MoS_2_/GR	One-pot solvothermal	RhB degradation 10 mg·L^−1^	150 W Xe lamp	DP of 80% in 80 min 3.9 times higher than TiO_2_ P25	Sheets/Particles	[34]
TiO_2_-CoS_2_/GR	Sonochemical and hydrothermal method	MB degradation 2.0 × 10^−5^ mol/L	8 W, halogen lamp 400–790 nm.	DP of >90% in 90 min	Sheets/Particles	[35]

^a^ QE: Quantum efficiency; ^b^ DP: Degradation percentage.
